# Development of the Fc-III Tagged Protein Expression System for Protein Purification and Detection

**DOI:** 10.1371/journal.pone.0044208

**Published:** 2012-08-29

**Authors:** Shan Feng, Enbing Tian, Lei Zhang, Qingtao Wang, Haiteng Deng

**Affiliations:** 1 School of Life Sciences, Tsinghua University, Beijing, China; 2 Beijing Chaoyang Hospital affiliated Capital Medical University, Beijing, China; Consejo Superior de Investigaciones Cientificas, Spain

## Abstract

In the present work, we developed the Fc-III tagged protein expression system for protein purification and detection. The Fc-III sequence encodes for a 13 residue peptide and this peptide is cyclized by disulfide bond formation when the fusion protein is expressed. The Fc-III-fusion proteins selectively bind to immunoglobulin Fc domains (IgG-Fc) expressed from *E. coli*. We showed the efficient purification of Fc-III tagged proteins by immobilized non-native IgG-Fc and the detection of the cellular locations of fusion proteins by fluorescent-conjugated IgG-Fc. Our results prove that Fc-III tagged protein expression system is a simple and efficient tool for protein purification and detection and is a useful addition to the biochemistry and proteomics toolbox.

## Introduction

Protein tagging involves genetically grafting a peptide sequence onto a recombinant protein. These fusion proteins have various applications in molecular biology such as affinity purification, protein detection, and epitope presentation [1]–[3]. Affinity tags that are appended to proteins are used to purify targeted proteins from their crude biological sources using affinity techniques. These include chitin binding protein (CBP), maltose binding protein (MBP), and glutathione-S-transferase (GST). The poly(His) tag is one of the most widely-used protein tag for protein purification with metal matrices [4]–[5]. Despite the broad applications of the existing affinity tags, it is difficult to decide on the best fusion system for a target protein of interest due to the diverse chemical natures of the target protein such as their stability, hydrophobicity, solubility and pI. Development of the new protein tags is important to provide a variety of tools for protein purification and detection.

**Figure 1:**
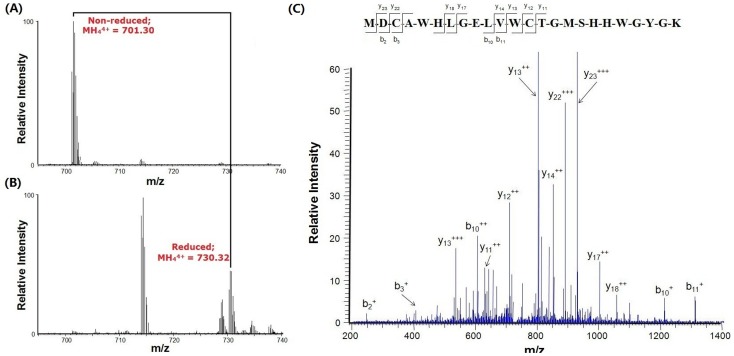
Comparison of reduced and non-reduced peptides containing Fc-III in MS spectra. (A) The MS spectrum of the tryptic peptide containing Fc-III peptide from trypsin digestion of the N-terminal Fc-III fused CA. The mono-isotope peak at m/z 701.30 matched to the quadruply charged peptide MDCAWHLGELVWCTGMSHHWGYGK. (B) The MS spectrum of the tryptic peptide containing carboxymethylated Fc-III peptide from trypsin digestion of the reduced and alkylated Fc-III-tagged CA The mono-isotope peak at m/z 730.32 matched to the quadruply charged peptide MDCAWHLGELVWCTGMSHHWGYGK with two carboxymethylated cysteines, (C) The MS/MS spectrum of quadruply charged peptide ion MH_4_^4+^ at m/z 730.32. The labeled peaks correspond to masses of y ions and b ions of the selected peptide.

In the present study, we engineered an Fc-III tagged fusion protein expression system. The Fc-III peptide is a mimic of Protein A, a commonly used affinity tag. The genetically expressed Protein A (PrA) fusion proteins from *Staphylococcus aureus* has a high affinity (10 nM) to the constant region (Fc) of immunoglobulin G (IgG) [6]. The large size of PrA may have adverse effects on the native conformation of the targeted protein and the binding to other proteins. Using phage display, several peptides have been identified to have high affinity to IgG-Fc [7]–[8]. One of the PrA like-peptides was named Fc-III [7]. The sequence of Fc-III peptide is DCAWHLGELVWCT-NH_2_, in which two Cys residues form a disulfide bond to make the cyclized peptide. The synthetic Fc-III peptide binds to the surface of the IgG-Fc domain by mimicking the protein-protein binding interface of PrA for the hinge region on the IgG-Fc. The affinity with a *K*_d_ value of 185 nM for the binding of the Fc-III peptide to IgG-Fc depends on both the Cys-Cys disulfide bridge and the C-terminal amide group [8]. An elegant application of Fc-III peptides was developed by Strambio-de-Castillia *et al.* to competitively displace affinity-purified Protein A fusion proteins and protein complexes from IgG-Sepharose with biotinylated Fc-III [9]. The other potential applications of synthetic Fc-III or Fc-III like peptides were to detect or purify human IgG molecules [10]–[11].

## Materials and Methods

### Plasmid Construction

The gene of CA (carbonic anhydrase) was a gift from Zhou HM’s laboratory at Tsinghua University and was cloned into the expression vector pET28a (Novagen). The primers of the inserting Fc-III peptide were the followings: (1) N-terminal insertion (F5: GACTGTGCATGGCATCTTGGAGAACTCGTATGGTGTACT GGAATGTCCCATCACTGGGG; r3: CATGGTATATCTCCTTCTTAAAGTTAAAC), and (2) C-terminal insertion (F5: GACTGTGCATGGCATCTTGGAGAACTCGTATGGTGTACT GAGCACTGAGATCCGGCTGC; r3: GAGTTTGAAGGAAGCTTTGATTTGCCTG). The codons of the Fc-III peptide are underlined. The gene of CK (creatine kinase) was bought from *Addgene*, and was cloned into pET21a (Novagen). Various primers were used to construct HisTag fused CK, N-terminal Fc-III tagged CK and C-terminal Fc-III tagged CK. The gene of CD38 was cloned from the mRNA by RT-PCR from the Raji cell line (purchased from ATCC), which was then sub-cloned into eukaryotic plasmid pcDNA3.1B (Invitrogen). The Fc-III peptide was added onto the N-terminus of CD38, and the primers are the followings: F5: ATGGACTGTGCATGGCATCTTGGAGAACTCGTATGGTGTACTGCCAACTGCGAGTTCAGCC; and r3: AAGCTTAACTAGCCAGC.

### Protein Expression and Purification

CA-II, Fc-III tagged CA-II, IgG-Fc and different CK proteins were expressed in *E. Coli* BL21(ED3)-*pLysS* strain (Stratagene, Heidelberg, Germany) and purified. Briefly, for Fc-III tagged CA and CK, the cell lysates were incubated with IgG-beads (GE Healthcare) or immobilized IgG-Fc sepharose beads for 1 hour at 4°C, and were eluted with HAc-NH_4_Ac (pH3.4); for other His-tagged proteins, Ni-FF (Qiagen) columns were used, and proteins were eluted with 250 mM imidazole in PBS (pH8.0). For biochemical analysis, the samples were dialyzed and loaded onto Sephacryl S300 column. The purity of the recombinant protein was characterized by SDS-PAGE. All the protein concentrations were determined using Bradford method with BSA as the standard.

**Figure 2:**
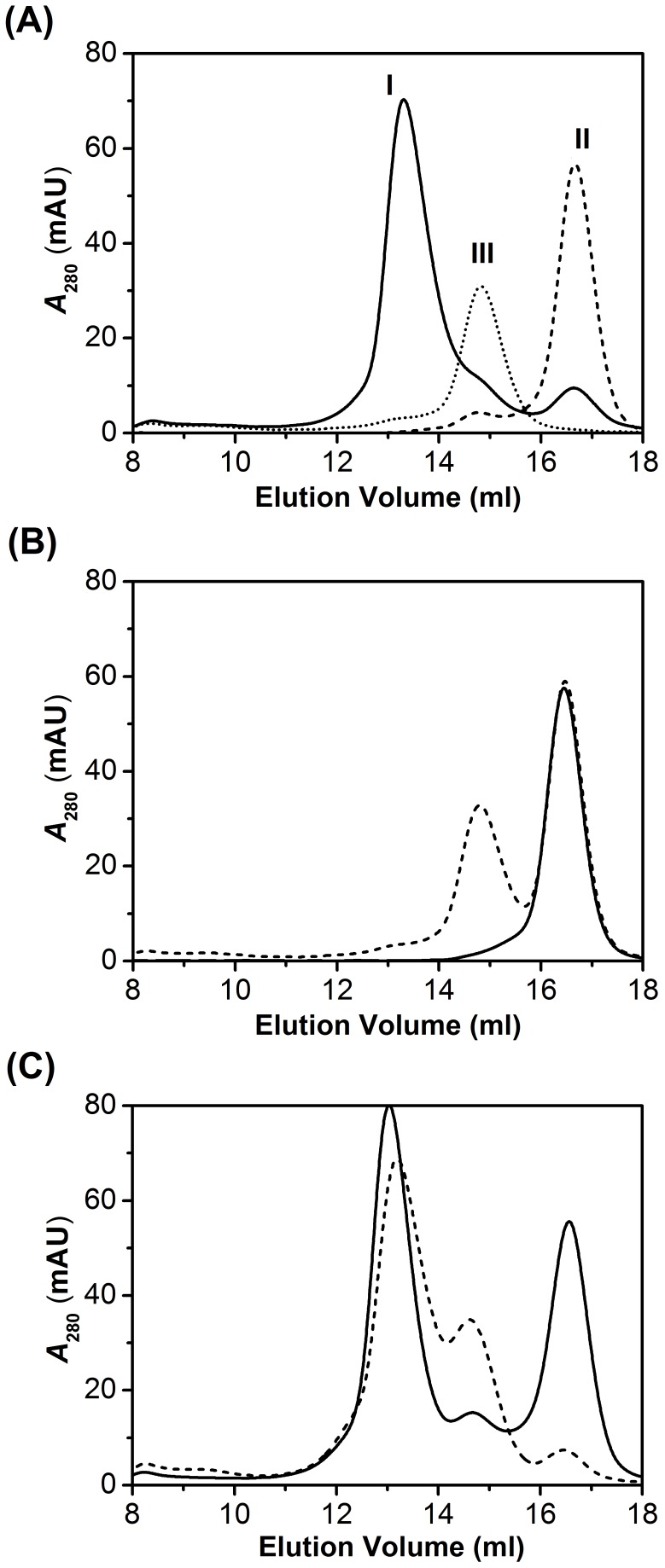
SEC analysis of the interaction between CA or Fc-III-tagged CA with IgG-Fc expressed from *E. coli*. (A) Peak I (solid line) represents the complex formation from incubation of 10 µM Fc-III-tagged CA with 5 µM IgG-Fc of at room temperature for half an hour. Peaks II and III (dashed and dotted line) represent the elution profiles of the Fc-III-tagged CA at 10 µM and the IgG-Fc at 5 µM, respectively. (B) the SEC profile (solid line) of CA alone at 10 µM and the SEC profile (dashed line) for the mixture of 10 µM CA and 5 µM IgG-Fc. No complex formation was observed. (C) The SEC profile (solid line) represents the complex formation from incubation of 20 µM Fc-III tagged CA with 5 µM IgG-Fc at room temperature for half an hour. The SEC profile (dashed line) represents the complex formation from incubation of 10 µM Fc-III-tagged CA with 10 µM IgG-Fc at room temperature for half an hour. 100 µl of each sample was injected into Superdex HR200 column.

### LC-MS/MS Analysis and Data Processing

Protein bands of interest were excised from the SDS PAGE and digested by trypsin with or without prior reduction and alkylation in 50 mM ammonium bicarbonate at 37°C overnight. The peptides were extracted twice with 1% trifluoroacetic acid in 50% acetonitrile aqueous solution for 30 min. The extractions were then centrifuged in a speedvac to reduce the volume.

For LC-MS/MS analysis, the digestion product was separated by a 65 min gradient elution at a flow rate 0.250 µL/min with the EASY-nLCII™ integrated nano-HPLC system (Proxeon, Denmark) which is directly interfaced with the Thermo LTQ-Orbitrap mass spectrometer. The analytical column was a home-made fused silica capillary column (75 µm ID, 150 mm length; Upchurch, Oak Harbor, WA) packed with C-18 resin (300 A, 5 µm, Varian, Lexington, MA). Mobile phase A consisted of 0.1% formic acid, and mobile phase B consisted of 100% acetonitrile and 0.1% formic acid. The LTQ-Orbitrap mass spectrometer was operated in the data-dependent acquisition mode using the Xcalibur 2.0.7 software and there is a single full-scan mass spectrum in the Orbitrap (400–1800 m/z, 30,000 resolution) followed by 20 data-dependent MS/MS scans in the ion trap at 35% normalized collision energy. The MS/MS spectra from each LC-MS/MS run were searched against the selected database using an in-house Mascot or Proteome Discovery searching algorithm.

### Size Exclusion Chromatography (SEC) Analysis

All the protein samples were dialyzed into 50 mM PBS (pH 7.8). For protein complex detection, Fc-III tagged or His-tagged CA were incubated with IgG-Fc for half an hour at room temperature, and then samples were centrifuged at 13 000 g and 100 µl sample was injected onto Superdex HR200 column and analyzed with the ÄKTA purifier 10 (GE Healthcare).

**Figure 3:**
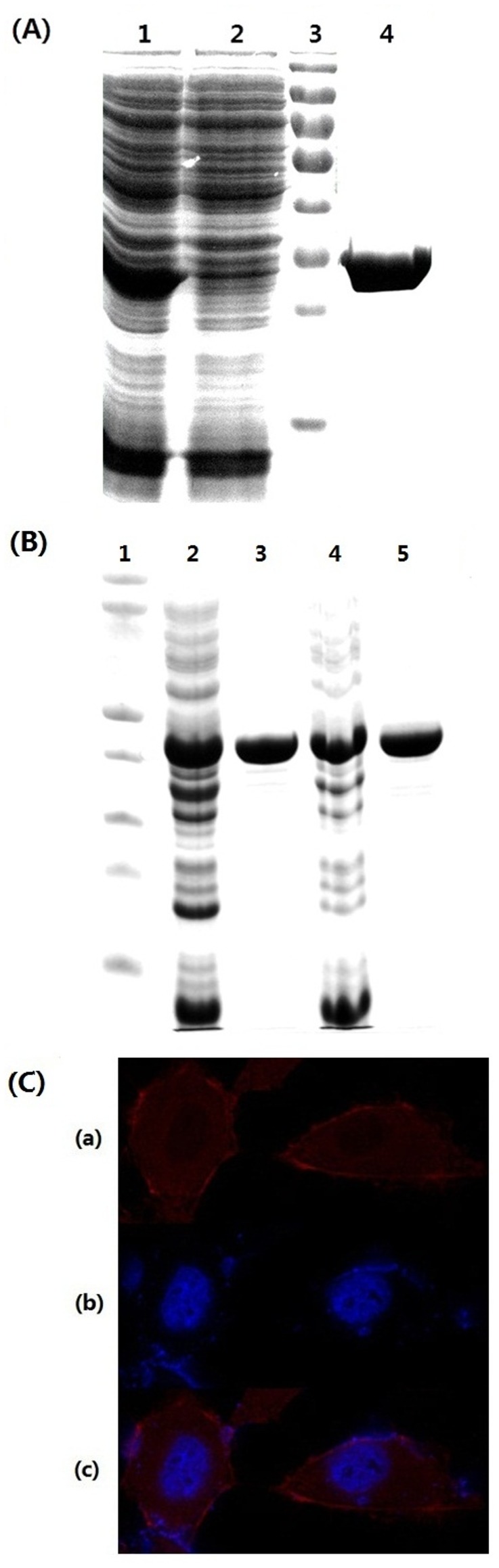
Applications of Fc-III fusion system for protein purification and detection. (A) The image of the SDS-PAGE of purification Fc-III-tagged by immobilized IgG-Fc Sepharose beads. Lane 1, whole cell extract; Lane 2, the unbound fraction; Lane 3, molecular weight marker; Lane 4, the eluents from immobilized IgG-Fc Sepharose beads. (B) The image of the SDS-PAGE of purification CKs. Lane 1, molecular weight marker; Lane 2&3, purification of N-terminal Fc-III-tagged CK; Lane 4&5, purification of C-terminal Fc-III-tagged CK. (C) Immunofluorescence detection of Fc-III tagged CD38 expressed in HUH-7 cell by Rhodamine B-conjugated Ig-Fc. (a) The image of Rhodamine B-stained CD38. (b) The image of the Hoechst 33342 stained HUH-7 nuclei. (c) The merged images from (a) and (b).

### Immuno-fluorescence

Human liver carcinoma HUH-7 cells (purchased from ATCC) were cultured on coverslips at 37°C in DMEM medium with 10% FBS under 5% CO_2_ atmosphere. After transfected with CD38 recombinant plasmid for 24 h, cells were fixed with 4% formaldehyde and permeabilised in 0.2% Triton X-100. The fixed cells were washed with PBS for three times, and then treated with 0.04 mg/ml Rhodamine B labeled IgG-Fc. Cells were stained with 1 µg/ml Hoechst 33342 for cell nucleus. After the washes, samples were analyzed by Zeiss LSM 710 (Carl Zeiss) Confocal Microscope with excitation wavelengths for Hoechst 33342 and Rhodamine B.

### Protein Quantitation by TMT Labeling

The purification efficiency was quantified by chemical labeling with TMT reagents (Thermo, Pierce Biotechnology, Rockford, IL) according to the manufacture’s instruction [12]. Briefly, the TMT labeling reagents were dissolved in anhydrous acetonitrile; carefully add the TMT label reagent to each digestion products; incubate the reaction for 1 hour at room temperature; use hydroxylamine to quench the reaction. The TMT-labeled peptides were desalted using the stage tips. The TMT^2^-126 reagent was used to label the tagged proteins binding to the affinity matrix, and TMT^2^-127 reagent was for the proteins left in the flow-through fraction. The ratio of the TMT^2^-126 labeled peptide to the TMT^2^-127 labeled peptides was calculated by the Proteome Discovery Software. The relative contribution from ^13^C isotope was deducted based on the manufacture’s protocol.

**Figure 4:**
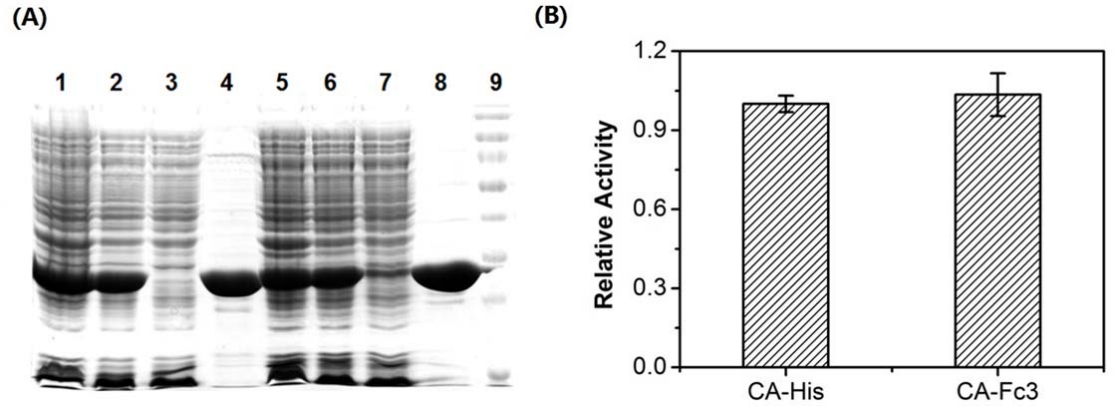
Comparison of protein expression and activities between His-tagged- and Fc-III tagged CA. (A) Lane 1, proteins containing His-tagged CA from crude whole cell lysate; Lane 2, proteins containing His Tagged CA from clear supernatant; Lane 3, proteins containing His-tagged CA from the flow-through fraction; Lane 4, the eluates of His-tagged CA from Ni-FF beads; Lane 5, proteins containing Fc-III tagged CA from crude whole cell lysate; Lane 6, proteins containing Fc-III tagged CA from clear supernatant; Lane 7, proteins containing Fc-III tagged CA from the flow-through fraction; Lane 8, the eluate of Fc-III tagged CA from the IgG-Fc beads; Lane 9, molecule maker. (B) Enzymatic activities of His-tagged CA and Fc-III tagged CA. The absorbance measurement was carried out at 348 nm wavelength and at 25°C in triplicate. Concentrations of both proteins were 1.0 mg/ml, respectively.

### CA Activity Measurement

The CA activity was determined using the Esterase activity assay as described by Pocker *et al* [13]. Briefly, 1 mM p-nitrophenyl acetate as the substrate was incubated with CA, the absorbance of the reaction mixture was monitored at 348 nm, and the reaction rate was calculated.

## Results and Discussion

To generate Fc-III fusion protein, we appended the DNA sequence of Fc-III (GACTGTGCATGGCATCTTGGAGAACTCGTATGGTGTACT) onto either the 5′ or 3′ end of the human *carbonic anhydrase* II (hCAII) that was cloned into pET-28a plasmid. CA has been widely used as the model system to study protein folding and enzymatic reaction. CA is broadly distributed among most of the tissues of animals and green plants and reversibly catalyzes the rapid interconversion of CO_2_ and H_2_O to HCO_3_^−^ and H^+^. The plasmid was introduced into *E. coli* T7 protein expression system for protein expression. The expressed protein was separated on the 1D SDS PAGE and a gel band corresponding to the expressed 32 kD Fc-III fusion proteins was observed. For finding out if the Fc-III in the fusion protein was in its cyclized form, the gel band was excised and digested with or without reduction and alkylation. The mass spectra of the tryptic peptides containing Fc-III are displayed in Figure 1. A peak at *m/z*701.30 in Figure 1(A) matched to the quadruply charged peptide ion with cyclized Fc-III that disappeared after reduction with DTT and alkylation with iodoacetamide, and instead a peak at *m/z* 730.32 corresponding to the carboxymethylated Fc-III-containing peptide ion was observed in Figure 1(B). The fragments of the precursor ions at *m/z* 730.32 precisely matched to the b ions or y ions of the carboxymethylated Fc-III-containing peptide. This shows that the expressed fusion protein has a cyclized Fc-III peptide.

Previous studies showed that cyclized Fc-III peptide as the mimic of PrA or PrG tightly binds to the hinge region on the Fc fragment of human IgG. Using the IgG immobilized sepharose beads, we were able to affinity purify the Fc-III fusion protein for carbonyl anhydrase (data not shown). To evaluate the binding of the Fc-III tagged fusion protein to *E. coli* expressed non-native IgG-Fc that does not have the glycosylation, we cloned and expressed the Fc fragment of human IgG in *E. coli*. The *E. coli* expressed IgG-Fc was incubated with the Fc-III fusion protein at several molar ratios and mixtures were analyzed using size-exclusion chromatography (SEC). Figure 2(A) shows the elution profile of the reaction products. The peak I (solid line) corresponds to the stable complex of IgG-Fc and Fc-III tagged CA. However, the complex was not formed when mixing CA and IgG-Fc as shown in Figure 2(B), indicating that the complex formation is based on the interaction of Fc-III and IgG-Fc. The stoichiometry of the complex is about 1 to 1 in their monomer forms as demonstrated in Figure 2(C), in which the change of the molar ratio of two reactants does not have effects on the complex formation. The *E. coli* expressed IgG-Fc is a homodimer as shown in Figure S1. These results showed the recombinant Fc-III fusion protein tightly binds to the recombinant IgG-Fc fragment to form a heterotetramer.

Unlike PrA or other binding proteins, Fc-III is a short peptide with 13 amino acid residues. It is expected that Fc-III peptide will not affect the native conformation and the solubility of the targeted proteins. Using reduced and non-reduced PAGE as shown in the Figure S1, we found that the inter-chain disulfide bond formation is at a minimum for the Fc-III tagged protein. In contrast, most *E. coli* expressed IgG-Fc is a dimer formed by inter-chain disulfide bond, in consistent with SEC analysis (Figure 2). To demonstrate that the recombinant IgG-Fc can be applied to purification of Fc-III tagged proteins, we have purified and immobilized the recombinant IgG-Fc onto agarose beads through Schiff base reaction. Incubation of the crude lysates of *E. coli* that expressed Fc-III tagged CA and immobilized IgG-Fc Sepharose beads leads to retention of the Fc-III tagged CA onto the beads as shown in Lane 2 in Figure 3(A). The Fc-III tagged CA can be fully recovered by acidic elution as shown in Lane 4 in Figure 3(A). Using a similar approach, we also purified Fc-III tagged CK (creatine kinase). Unlike CA, CK has a complex structure, which contains 2 subunits and totally 8 free cysteines. The Fc-III tag was fused into either the N-teminus or the C-teminus of CK. As shown in Figure 3(B), both forms of the recombinant proteins were easily purified from the cell lysates. This shows that the Fc-III tag is applicable to purification of the large and multiple-Cysteine-containing proteins. These results indicate Fc-III fused system is a tool of choice for protein purification.

Immuofluorescence is a power tool which uses the highly specific binding of an antibody to its antigen to detect specific proteins within the cell. Taking the advantage of the tight binding of Fc-III with the non-native IgG-Fc, we may detect the Fc-III tagged protein with fluorescent-labeled IgG-Fc expressed from *E. coli*. As proof-of-principle, we expressed Fc-III tagged CD38 in HUH-7 cell line. CD38 also known as cyclic ADP ribose hydrolase, is localized on the surface of many immune cells and plays important roles in synthesis and hydrolysis of cyclic ADP-ribose, signal transduction, and cell adhesion [14]. After cell fixation on a glass slide, Rhodamine B-labeled IgG-Fc generated by a reaction of Rhodamine B succinimidyl ester with IgG-Fc (see Figure S2) was added onto the cells. As shown in Figure 3(C), the red fluorescence was observed on the membrane of HUH-7 cells labeled by Rhodamine B-conjugated IgG-Fc, indicating the localization of CD38 on the plasma membrane of HUH-7 cells.

We have also evaluated the effects of Fc-III tag on the expression level, the purification efficiency, and the activity of the targeted protein. Compared to His-tagged CA, fusion with Fc-III tag does not have adverse effects on the expression level of CA, as shown in Lane 1 & 5 of Figure 4(A), in which the same amount of proteins from the whole cell lysates were loaded. The purity of Fc-III tagged CA binding to IgG-Fc beads is higher than that of His-tagged CA binding to Ni-FF as shown in Lane 4 & 8 of Figure 4(A). Using the quantitative proteomics with TMT labeling, we calculated the ratios of the affinity-purified CA to the unbound CA based on the MS/MS spectra of 10 TMT-labeled tryptic peptides from CA. The MS/MS spectra are displayed in the Figure S3. We estimated the purified protein yields are 92% for HisTag and 88% for the Fc-III tagged CA, respectively. The Fc-III tag does not change the enzymatic activity of CA, as shown in Figure 4(B). The Fc-III tagged and His-tagged CA exhibited the similar activities to p-nitrophenyl acetate.

In summary, we demonstrated that the expressed Fc-III tagged proteins have a cyclized Fc-III ring formed by disulfide linkage. The Fc-III fusion protein tightly binds to non-native IgG-Fc expressed from *E. coli*. This fusion system allows affinity purification of targeted proteins by immobilized IgG-Fc. We also illustrated that the CD38 fusion proteins were localized onto the plasma membrane in HUH-7 cells as detected by florescent-conjugated IgG-Fc fragment. Since Fc-III peptide has only 13 amino acid residues, it is expected that the Fc-III fusion system has advantages over the PrA tag and is a simple and efficient tool for protein purification and detection.

## Supporting Information

Figure S1 **1D SDS PAGE of expressed proteins under reduced (Lane 1–4) and non-reduced (Lane 6–9) conditions, respectively.** Lane 1&6 for His-tagged CA; Lane 2&7 for Fc-III-tagged CA; Lane 3&8 for IgG-Fc purified from *E. coli*; and Lane 4&9 for the mixture of Fc-III tagged CA and IgG-Fc. Lane 5 is the marker indicated the molecular weight. Noting that most expressed Fc-III tagged CA were monomers and most expressed IgG-Fc are dimers.
(TIF)

Figure S2 **Rhodamine B-labeled IgG-Fc was generated by a reaction of Rhodamine B succinimidyl ester with IgG-Fc.** To prove the labeling efficiency, in-gel fluorescence images of IgG-Fc labeled with rhodamine B succinimidyl ester was acquired with the exciting wavelength at 572 nm. Lane 1, 0.4 mg/ml IgG-Fc; Lane 2, 0.8 mg/ml IgG-Fc; Lane 3, 0.8 mg/ml IgG-Fc reacted with rhodamine B for 4 h; Lane 4, 0.4 mg/ml IgG-Fc reacted with rhodamine B overnight; Lane 5, 0.8 mg/ml IgG-Fc reacted with Rhodamine B overnight.
(TIF)

Figure S3 **The MS/MS spectra of 10 TMT-labeled tryptic peptides from CA.** Each MS/MS spectrum shows the relative intensity of two major fragments at m/z 126 and 127, corresponding to the TMT^2^-126-labeled and the TMT^2^-127-labeled peptides, respectively. The intensity ratio of these two fragments represents the relative quantity of the targeted protein on the affinity matrix and in the flow through fraction. The ^13^C contribution of TMT^2^-126 was used to calculate the purification efficiency. (A–J) 10 peptides from His-tagged CA; (A–J) 10 peptides from Fc-III tagged CA.
(TIF)
