# Supplementary figures and images for: Development of the Fc-III Tagged Protein Expression System for Protein Purification and Detection
Source: PLoS One. 2012 Aug 29;7(8):e44208. doi: 10.1371/journal.pone.0044208 (PMC3430635; doi:10.1371/journal.pone.0044208)

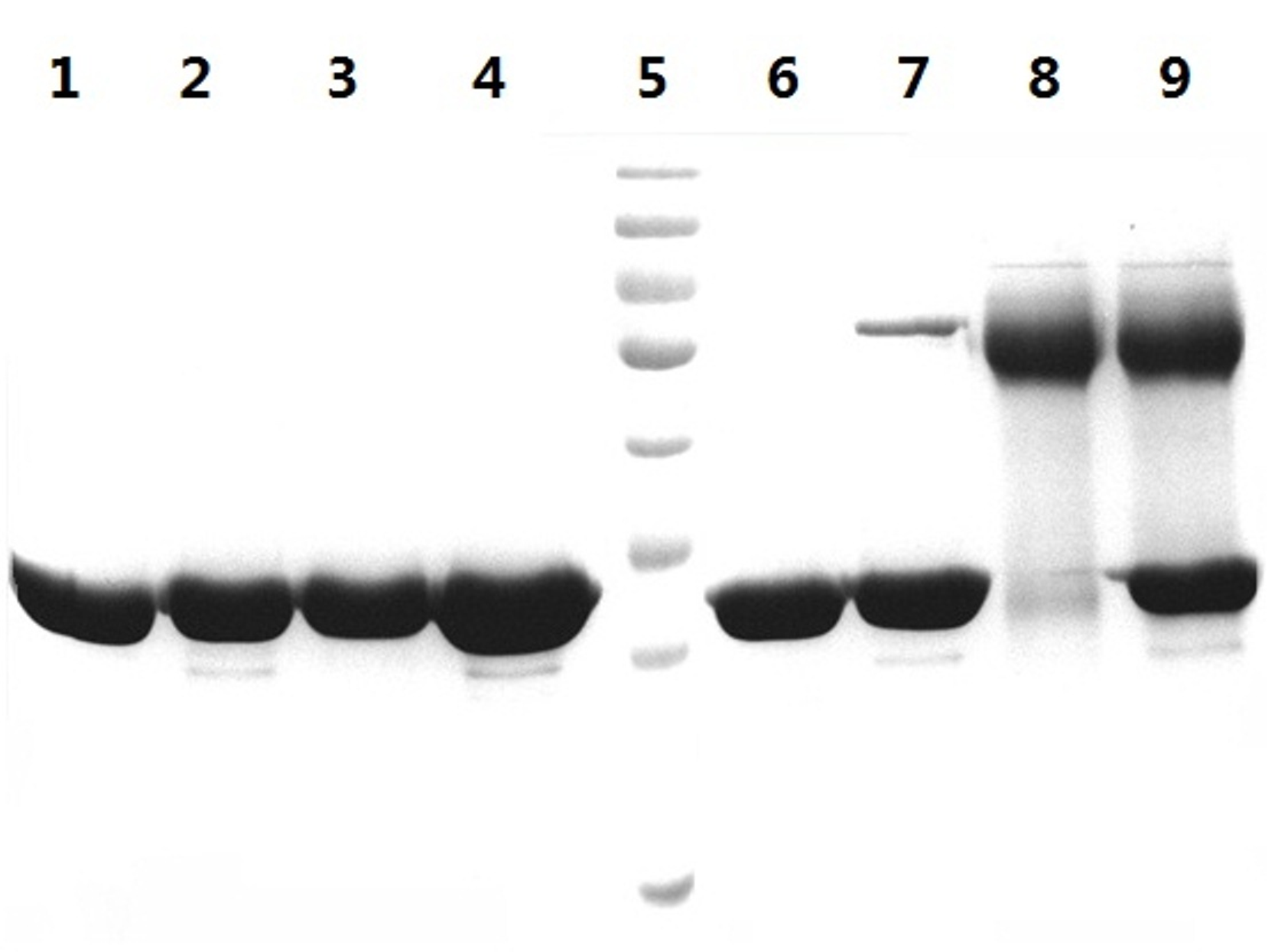

Supplement: Figure S1 — 1D SDS PAGE of expressed proteins under reduced (Lane 1–4) and non-reduced (Lane 6–9) conditions, respectively. Lane 1&6 for His-tagged CA; Lane 2&7 for Fc-III-tagged CA; Lane 3&8 for IgG-Fc purified from E. coli; and Lane 4&9 for the mixture of Fc-III tagged CA and IgG-Fc. Lane 5 is the marker indicated the molecular weight. Noting that most expressed Fc-III tagged CA were monomers and most expressed IgG-Fc are dimers. (TIF) [file pone.0044208.s001.tif]

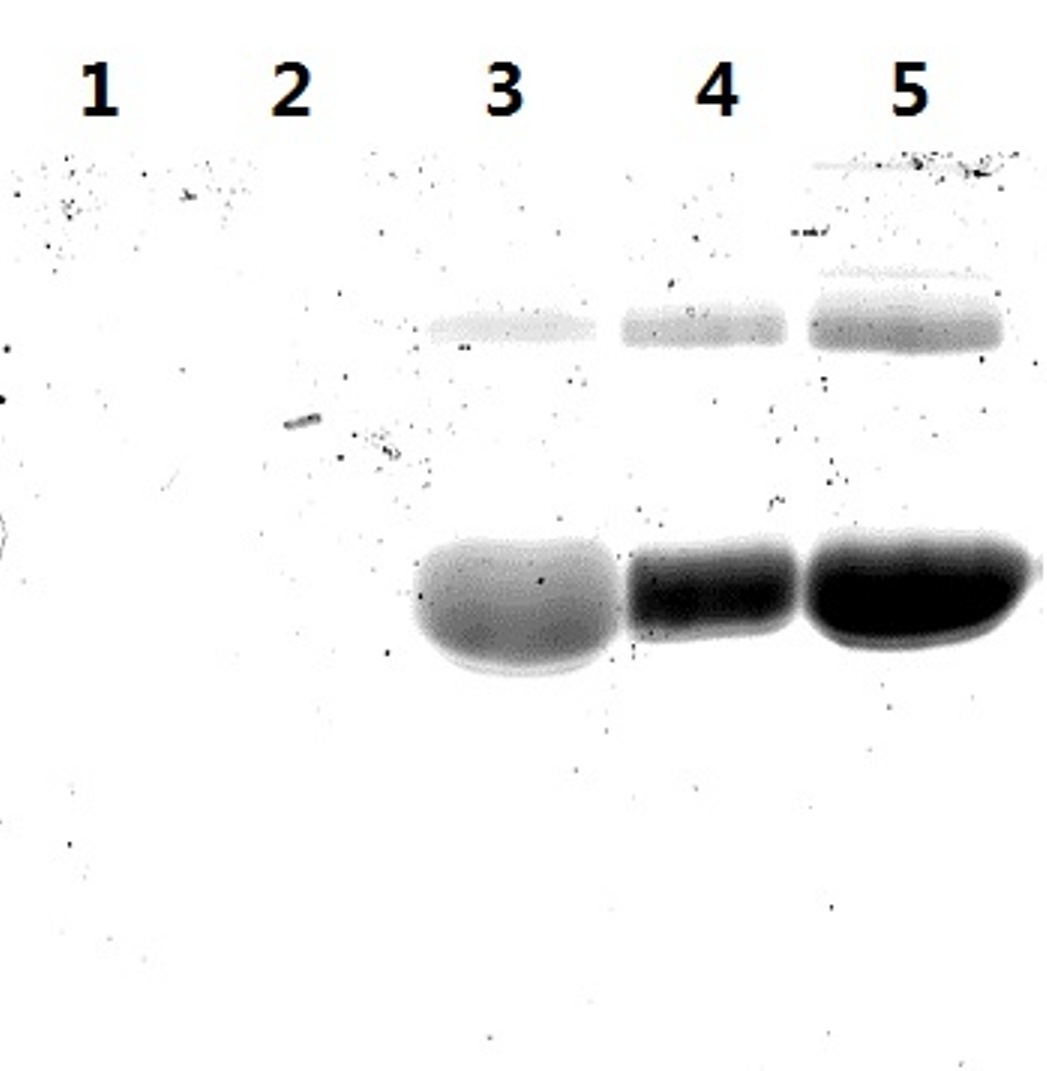

Supplement: Figure S2 — Rhodamine B-labeled IgG-Fc was generated by a reaction of Rhodamine B succinimidyl ester with IgG-Fc. To prove the labeling efficiency, in-gel fluorescence images of IgG-Fc labeled with rhodamine B succinimidyl ester was acquired with the exciting wavelength at 572 nm. Lane 1, 0.4 mg/ml IgG-Fc; Lane 2, 0.8 mg/ml IgG-Fc; Lane 3, 0.8 mg/ml IgG-Fc reacted with rhodamine B for 4 h; Lane 4, 0.4 mg/ml IgG-Fc reacted with rhodamine B overnight; Lane 5, 0.8 mg/ml IgG-Fc reacted with Rhodamine B overnight. (TIF) [file pone.0044208.s002.tif]

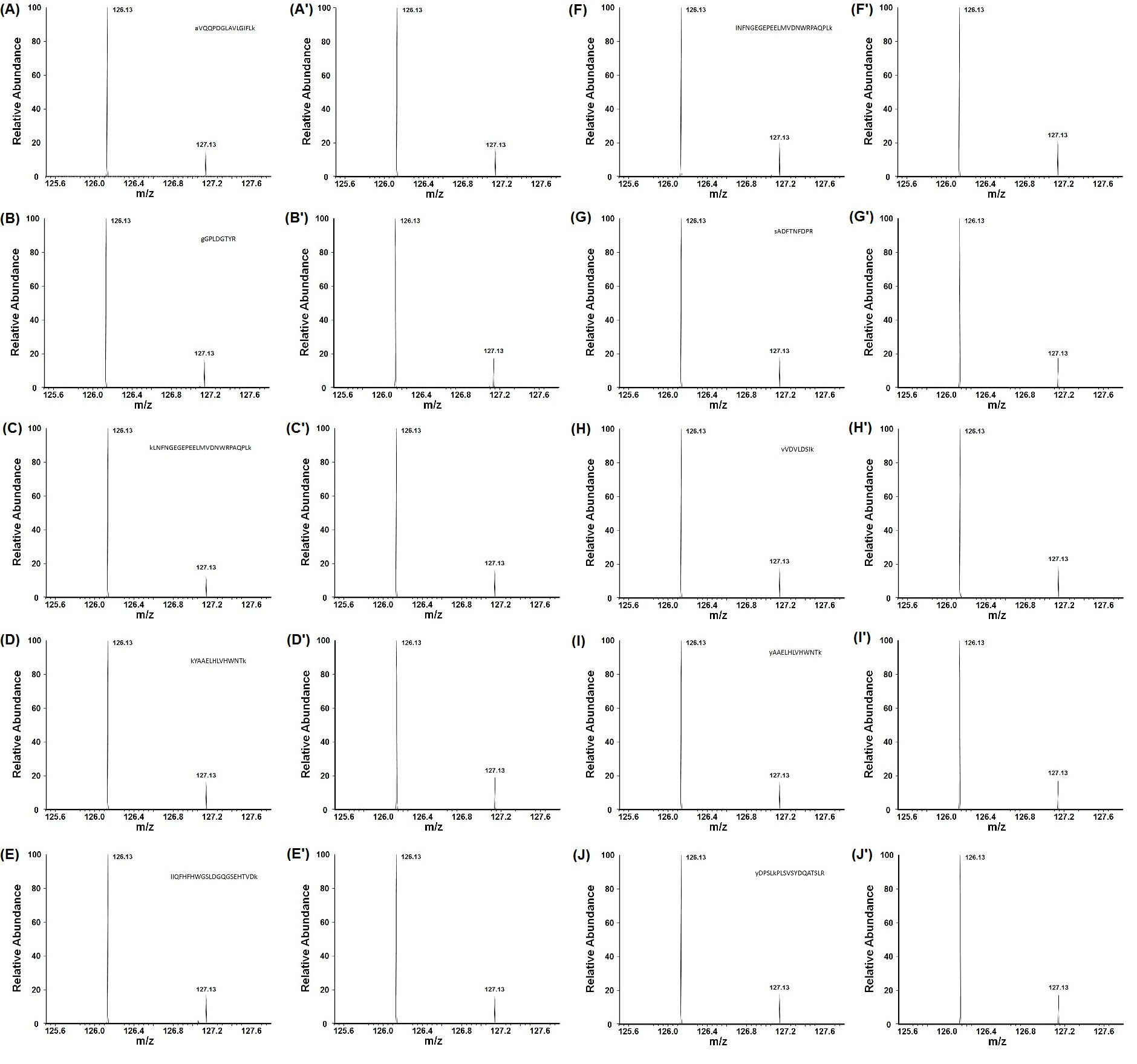

Supplement: Figure S3 — The MS/MS spectra of 10 TMT-labeled tryptic peptides from CA. Each MS/MS spectrum shows the relative intensity of two major fragments at m/z 126 and 127, corresponding to the TMT2-126-labeled and the TMT2-127-labeled peptides, respectively. The intensity ratio of these two fragments represents the relative quantity of the targeted protein on the affinity matrix and in the flow through fraction. The 13C contribution of TMT2-126 was used to calculate the purification efficiency. (A–J) 10 peptides from His-tagged CA; (A–J) 10 peptides from Fc-III tagged CA. (TIF) [file pone.0044208.s003.tif]
